# Further Evidence That N2pc Reflects Target Enhancement Rather Than Distracter Suppression

**DOI:** 10.3389/fpsyg.2017.02275

**Published:** 2018-01-04

**Authors:** Chaojie Li, Qiang Liu, Zhonghua Hu

**Affiliations:** Research Center of Brain and Cognitive Neuroscience, Liaoning Normal University, Dalian, China

**Keywords:** N2-posterior-contralateral (N2pc), attention, event-related potential (ERP), visual search, pop-out

## Abstract

The N2-posterior-contralateral (N2pc) component is an index in the domain of event-related potentials for exploring the underlying mechanism of visual-spatial attention. It has been disputed whether the attentional selection reflected by N2pc is primarily due to distracter suppression or target enhancement processes. We addressed this controversy by combining the pop-out item and the target feature, and instructed participants whether the pop-out item included the target feature. Thus, in a visual search task, bilateral visual stimuli including a pop-out item and three distractors were displayed simultaneously. The pop-out detection was analyzed under varying two factors: (a) pop-out item as a target or non-target (b) the distractors containing a target feature or non-target feature. Although all conditions had a salient effect on behavioral performance, the reliable difference of N2pc existed only between the target condition and the non-target condition. These results provided strong support for the hypothesis of target enhancement processes.

## Introduction

The event-related potential (ERP) components are generally applied to explore the specific neural process and disclose the underlying mechanism of brain function with its high temporal resolution when a certain type of task is manipulated. Previous studies have been certified on a series of issues that the ERP components could reflect the mechanism of attention (Luck et al., 2000). For example, the ERP component discussed in the present study, which was labeled as N2-posterior-contralateral (N2pc), is closely related to spatial attention, reflecting the process of attentional distribution to the current task-related stimulus (Luck and Hillyard, 1994a,b; Eimer, 1996, 1998; Luck et al., 1997; Luck and Ford, 1998; Woodman and Luck, 1999; Brisson and Jolicoeur, 2007b; Brisson et al., 2007; Mazza et al., 2009; Töllner et al., 2011; Zhao et al., 2011; Grubert and Eimer, 2016; Liu et al., 2016).

N2pc means that there is a more negative amplitude in the contralateral posterior electrodes to the target compared with the ipsilateral posterior region to the target. Namely, in the case of the left posterior electrode sites, the targets appeared in right visual field could evoke a more negative amplitude than appeared in left visual field. Nevertheless, in the case of the right posterior electrode sites, the condition is opposite. The latency of the N2pc is typically 180–350 ms after the appearance of unilateral stimulation. Brain source analyses showed that this component could arise from lateral portions of the extrastriate and infero-temporal visual areas (Hopf et al., 2000, 2004).

A growing number of studies have demonstrated that the N2pc component could be considered as an index of covert visual-spatial attention; however, attention is a broad umbrella term that includes diversiform types of processes (for reviews, see Luck and Gold, 2008). There is still controversy about the essential process reflected by N2pc. On the one hand, Luck and Hillyard (1994b) implied that the N2pc mirrored the inhibition of neural activity caused by irrelevant or conflicting items in visual search processing. It was in accordance with the existence of a spatial filtering mechanism (LaBerge and Brown, 1989). It demonstrated that attention served as a filter which constituted a gradient of inhibition around a selected location. This point is supported by evidence that N2pc was only elicited by a target item surrounded by competing distractors (Luck and Hillyard, 1994b). Compared with the “easy non-targets” condition, there existed a robust N2pc in the “difficult non-targets” even though there was no target. For example, if the small blue vertical bar was the target, the large blue vertical bar was a difficult non-target and the large green horizontal bar was an easy non-target. In addition, the amplitude of N2pc was increased when the number of distracters nearby the target was increased from 1 to 3 (Luck et al., 1997) and from 3 to 19 (Mazza et al., 2009).

On the other hand, the view of suppressing competitive information has been challenged by the evidence that N2pc can also be elicited when only one target item was presented on one side of the visual field together with one non-target on the other side of the visual field (Eimer, 1996; Wascher and Wauschkuhn, 1996; Brisson and Jolicoeur, 2007a; Brisson et al., 2007). Eimer (1996, 1998) argued primarily that N2pc was more likely to reflect the top–down neural mechanism, which was sensitive to task-related features rather than the interferential stimulus. What is more, Hickey et al. (2009) manipulated the locations the stimuli array contained two objects, one set on the vertical midline and the other set to the unilateral side of fixation. One of the objects was a square or a diamond, and the other was a short or long horizontal line. The N2pc component was observed contralateral to the target when the distracter was on the vertical midline. It was proposed that N2pc reflected a process that enhanced the cortical representation of the target rather than a process that filtered distracters. However, just as Luck (2012) mentioned that the essential difficulty in estimating the controversial hypothesis was that the independent means of filtering process could not be manipulated effectively. Namely, it was uncertain whether the hypothesized filtering process was present or absent under a given set of experimental conditions.

The purpose of the present study was to further investigate the underlying process of attentional selection according to the interpretation of N2pc. In the present experiment, a green letter as a pop-out item was presented together with three blue letters as distractors. The participants were asked to detect whether the pop-out item was the target. Because the target item must meet two criteria, one of the criteria was the pop-out item and the other was the target feature (the letter “T”). Only if the item met the two criterions could participants made a response. That is, the pop-out item was the necessary but not sufficient condition for the target. Accordingly, this process contained two kinds of attentional components, the bottom–up attentional capture and the top–down attentional sets. Those two parts contributed to the amplitude of N2pc. Specifically, the top–down attentional set included the target feature “T,” whereas the green item as the discrepancy was easy to capture the attentional resource, which referred to the bottom–up process. Previous studies have shown that, in the visual search task, it was easier to search for a highlighted item than to search for a singleton (Eimer and Kiss, 2010).

The N2pc amplitude was coded by manipulating two variables: (a) pop-out item existed as a target or non-target (b) the distractors existed with a target feature or without the target feature. We assumed that subjects would first attend to the pop-out item followed by distinguishing whether the pop-out item was the target. Consequently, there were four conditions to consider the processes of target enhancement or distracter suppression. Hypothetically, if N2pc was elicited by the suppression of neural activity produced by surrounding distracters, the pop-out item would elicit the N2pc amplitude anyway regardless of whether the pop-out item was the target. Furthermore, the distractors with a target feature might elicit a more negative N2pc than the distractors without a target feature. But if N2pc reflected the attentional selection of task-relevant features, the pop-out item as a target would elicit the more salient N2pc amplitude than the pop-out item as a non-target. Moreover, no matter distractors existed with a target feature or not, it would not affect the N2pc amplitude.

## Materials and Methods

### Participants

Twelve undergraduate students (seven females, mean age = 21.25, range: 19–22) participated in the study as paid participants. Each of the participants was a native Chinese speaker who was right-handed and normal or correct-to-normal vision. All subjects signed informed consent before the experiment.

### Stimuli

**FIGURE 1:**
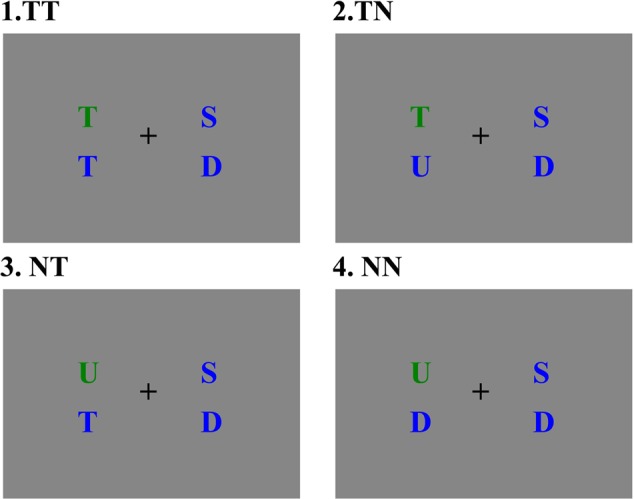
Four stimulus types, (1) target at pop-out position with a distracter containing the target letter feature; (2) target at pop-out position with a distracter without the target letter feature; (3) non-target at pop-out position with a distracter containing the target letter feature; (4) non-target at pop-out position with a distracter without the target letter feature.

The stimuli were presented on a 17-inch CRT monitor screen, 67 cm away from the participants. The display has a screen resolution of 1024 × 768 and a screen refresh rate of 85 Hz. The stimulus consisted of four colored uppercases with a visual angle of 0.7°, including a green uppercase (pop-out item) and three blue uppercases (distractor items). Two letters were presented on the left side of the fixation and the other two letters were presented on the right side of the fixation, with an upper part and a lower part for each side on a gray background (see **Figure 1**). The horizontal distance between each side of the display and the central fixation point was 3°.

In the visual search, the target letter is the green letter “T,” which appeared randomly at one of the four positions in the visual display. Thereby the target item in this study contained two features, namely a color feature “green” and a letter feature “T.” To explore the effect of distractors on N2pc, the blue distractors which appeared on the same side with the pop-out item were manipulated into two categories: containing a target feature “T” or a non-target feature (“D,” “U,” or “S”). Therefore, the experiment contained four conditions: target item at pop-out position accompanied with a distracter item containing a target letter feature (TT), target item at pop-out position accompanied by a distracter item without target letter feature (TN), non-target item at pop-out position accompanied by a distracter item containing a target letter feature (NT), non-target item at pop-out position accompanied by a distracter item without the non-target letter feature (NN).

### Procedure

The participants were given instructions on the task in a soundproofed room. Participants needed to distinguish whether the pop-out letter was the target letter “T” as soon as possible. At the start of each trial, a fixation was played randomized for 1000–1300 ms with a gray background and then the detected interface was displayed for 200 ms. If the pop-out item was the target letter “T,” a response was triggered by pressing the ‘1’ key using index finger. While if the pop-out item was not a target letter “T,” a response was triggered by pressing the ‘2’ key using the middle finger. The next trial would not begin until the participant reacted. Thus, in the course of the experiment, the participants were required to react both quickly and accurately. In addition, they were required to control head movement and pay attention to the central fixation.

The pop-out items emerged in all trials and the target existed at the pop-out position with half of the times. In order to keep spatial balance of visual display, the pop-out item would appear at each of the four positions randomly. Thereby, combined with the four types of stimulus conditions, there were totally 16 conditions for one round. The trials in one round were presented randomly to the participants. Each participant should complete six blocks containing six rounds in each block. Thus, there were 576 trials in all in the whole experiment for each participant.

### Electrophysiological (EEG) Recording and Analysis

The electrode cap produced by Brain Products GmbH was used to collect the electroencephalograms (EEGs) which was composed of 64 scalp sites using tin electrodes with a sampling frequency of 500 Hz. The impedance of each electrode was less than 5 kΩ. These electrodes and the left earlobe electrode were recorded with a right-earlobe reference. The ERP waveforms were then re-referenced offline to the average of the left and right mastoids. Moreover, bipolar horizontal and vertical electrooculograms (EOGs) were recorded simultaneously to monitor eye movements. The EEG and EOG were amplified by a 0.01–100 Hz bandpass for offline analysis. The artifacts of eye movement were rejected offline. Only the correct trials were analyzed. Besides, those trials that exceed the borderline of the eyeblinks (vertical EOG amplitude out the range of ±100 μV and Horizontal EOG amplitude out the range of ±25 μV) were deleted. On account of the subtle difference between the bilateral target positions it was difficult to eliminate by the artifact rejection of horizontal EOG. We divided the trials into two conditions that the target was right or left visual field, so that we could calculate the average difference recording by the HEOG electrodes respectively. Maximal deflections of all participants were less than ±3 μV (i.e., residual eye movement < 0.2°). With the completion of the data rejecting process, the retained trials of all participants were on average 91%.

The ERPs of each condition were averaged respectively and filtered digitally with a low-pass half-power cut-off frequency of 30 Hz. The averaged epoch for ERPs was 1000 ms including a 100 ms pre-stimulus baseline. The N2pc components were quantified on the basis of mean amplitudes obtained in the 210–290 ms time window at lateral posterior electrodes PO7 and PO8. N2pc was the grand-average waveforms calculated from the contralateral waves minus ipsilateral waves. Particularly, the ipsilateral waves contained the average waves of left-sided electrodes with the left-visual field target and right-sided electrodes with the right-visual field target. And the contralateral waves contained the average waves of left-sided electrode with the right-visual field target and right-sided electrode with the left-visual field target. We compared the N2pc effects among the four types in a 2 × 2 repeated-measures ANOVA [(pop-out class: target or non-target) × (distracter class: target feature or non-target feature)]. For all analyses, *p*-value was corrected for deviations according to Geisser–Greenhouse *F*-test.

## Results

### Behavioral Results

The average accuracy of all four cases was above 97%, and error trials were excluded from the analysis of response time (RT). The mean RTs for TT, TN, NT, and NN condition were 535.70 ± 43.51 ms, 547.47 ± 40.30 ms, 572.40 ± 49.43 ms, and 557.55 ± 55.54 ms, respectively. The RT data were analyzed using a 2 (pop-out class: target or non-target) × 2(distracter class: target feature or non-target feature) analysis of variance (ANOVA) with repeated measures over both factors. The results showed that there was a reliable main effect of pop-out class which suggested that participants needed more time to complete the task in non-target conditions [*F*(1,11) = 5.732, *p* = 0.038, $\eta_{p}^{2}$ = 0.364]. The main effect of distracter class was not salient [*F*(1,11) = 1.048.630, *p* = 0.328, $\eta_{p}^{2}$ = 0.059]. The interaction between pop-out class and distracter class was significant [*F*(1,11) = 25.101, *p* = 0.001, $\eta_{p}^{2}$ = 0.715]. Based on a simple main effects analysis followed: the RTs of the target-present and target-absent conditions were different significantly for the distracter with a target feature [*F*(1,11) = 12.091, *p* = 0.006, $\eta_{p}^{2}$ = 0.547] other than with a non-target feature [*F*(1,11) = 1.086, *p* = 0.322, $\eta_{p}^{2}$ = 0.098]. There were both a reliable main effect of distracter class for the target-present condition [*F*(1,11) = 17.200, *p* = 0.002, $\eta_{p}^{2}$ = 0.632] and for the target-absent condition [*F*(1,11) = 16.221, *p* = 0.002, $\eta_{p}^{2}$ = 0.619].

### ERP Waveform Analysis

**FIGURE 2:**
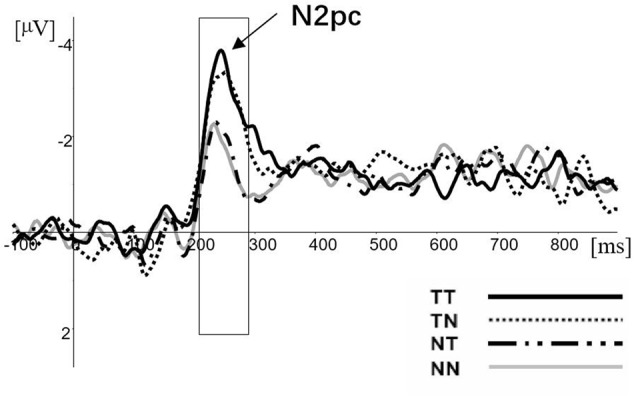
The N2pc difference waves for TT, TN, NT, and NN conditions at electrode sites PO7 and PO8.

The N2pc difference waveforms for the four conditions (TT, TN, NT, and NN) are illustrated in **Figure 2**. The mean amplitudes during the 210∼290 ms post-visual display time window were analyzed. Firstly, the results of paired-samples *t*-test indicated that each of condition evoked N2pc (compared with zero uV) significantly (TT: *t* = -3.924, *df* = 11, *p* = 0.002; TN: *t* = -3.498, *df* = 11, *p* = 0.002; NT: *t* = -3.185, *df* = 11, *p* = 0.009; NN: *t* = -2.930, *df* = 11, *p* = 0.014). Secondly, the mean amplitudes of N2pc were analyzed using a 2 (pop-out class: target or non-target) × 2 (distracter class: target feature or non-target feature) analysis of variance (ANOVA) with repeated measures over both factors. The main effect of target variable was significant [*F*(1,11) = 10.900, *p* = 0.007, $\eta_{p}^{2}$ = 0.852]. The main amplitude of N2pc for target conditions was found much more negative than for the non-target condition. However, the main effect of the distracter variable was not salient [*F*(1,11) = 0.691, *p* = 0.423, $\eta_{p}^{2}$ = 0.119], the interaction was not significant [*F*(1,11) = 1.883, *p* = 0.197, $\eta_{p}^{2}$ = 0.241].

## Discussion

The aim of the present study was to investigate the specific processes underlying N2pc. The behavioral data showed that when the target was presented, the distracter with a target feature elicited a faster response than the distracter without the target feature. However, when the non-target stimulus was presented, the distracter with a target feature elicited a slower response than the distracter without a target feature. These results indicated that participant’s behavioral performance of detecting the target varied depending on whether the distracters were involved with a target feature. In the target condition, the target-feature of distracter might facilitate the visual searching performance by enhancing or exposing the feature of the target. While in the non-target condition, the distracter with the target feature interfered with the visual search process, which induced participants to spend more time to verify the target feature in the array. These were consistent with Woodman and Luck’s conclusion that the content of working memory might facilitate or inhibit the processes of the visual searching task in a flexible manner (Woodman and Luck, 2007). What’s more, the target feature that appeared in the distracters item affected the RT, which indicated that the distracters could not be suppressed by attention, that is, the processes of attentional filter did not work.

In each condition, the amplitude of N2pc was significant (see **Figure 2**). Furthermore, the amplitude was not regulated by the distracters, although the distractors including a target feature had an effect on the response-level modulation. According to hypothesis of suppression, the N2pc reflects the neural activity which produced by suppression of distracter (Luck and Hillyard, 1994b). If so, the relationship between target and background would affect the amplitude of N2pc, such as the number of distracters and the distance and the similarity between target and distracters. However, the result had not shown a salient difference between NT and NN. Thereby, the current results did not support the hypothesis of suppression.

The only salient difference of N2pc in our result appeared between the target condition and the non-target condition. Combined with the behavioral and ERP results, we could better explain that N2pc was moderated by the processes of target enhancement rather than the distracter suppression. As the results showed, although the distracters with a target feature could influence the response time, it could not regulate the amplitude of N2pc. On the contrary, if N2pc had been moderated by attentional filter, there would have been no difference in the response time in each condition. In addition, it would regulate the amplitude of N2pc. Specifically, the bottom–up process of the pop-out item combined with the target feature could evoke a larger N2pc compared with the only pop-out item without the target feature. Previous studies have confirmed that the amplitude of N2pc was positively correlated with the degree of salience of the target item (Töllner et al., 2011; Zhao et al., 2011) and the difficulty of the task (Liu et al., 2016), which suggested that the amplitude of N2pc relates to attention resource allocation. Thus, the current results suggested that a target involved in more task-relative features would attract more attentional resources. Consequently, the current result provided strong support for the Eimer’s hypothesis that N2pc reflects attentional selection of target stimulus features.

In addition, we suggested that the following process led to an N2pc. When the visual array was presented to observers, the distinct color of pop-out item, regardless of the target feature or interference feature, would be exposed from other distracters and catch participants’ attention. Then the pop-out item was compared with the task-relevant feature kept in working memory. When the pop-outing item matched the target in working memory, a more negative waveform was elicited to reflect the matched target; if not, no additional reflection would appear.

## Conclusion

The four conditions of the visual search tasks were found in the N2pc component. However, the only salient difference in N2pc amplitude appeared between the pop-out item as a target and the pop-out item as an interferential non-target, regardless of whether the distractors contained a target-feature item or not. These results provided a strong support for the hypothesis that N2pc reflects target enhancement.

## Ethics Statement

This experiment was carried out in accordance with the recommendations of procedures and protocols approved by the Human Subjects Review Committee of Liaoning Normal University with written informed consent from all participants.

## Author Contributions

CL was responsible for the design of the experiment, data analysis, and paper writing. QL was responsible for writing the experimental program and analysis. ZH was responsible for the design of the experiment and editing the language of the article.

## Conflict of Interest Statement

The authors declare that the research was conducted in the absence of any commercial or financial relationships that could be construed as a potential conflict of interest.
